# SOI-Structured Piezoresistive Pressure Sensor with Integration of Temperature Sensor for Downhole Applications

**DOI:** 10.3390/s26072076

**Published:** 2026-03-26

**Authors:** José Mireles Jr., Abimael Jiménez, Ángel Sauceda

**Affiliations:** Institute of Engineering and Technology, Universidad Autónoma de Ciudad Juárez, 450 Avenida del Charro, Ciudad Juárez 32310, Mexico; jmireles@uacj.mx (J.M.J.); angel.sauceda@uacj.mx (Á.S.)

**Keywords:** micro-electro-mechanical system, pressure sensor, temperature sensor, finite element modeling, silicon on insulator, fabrication, MEMS characterization

## Abstract

Micro-electro-mechanical systems (MEMS) sensors offer the benefits of compact size, lightweight design, and low cost, which has led to widespread use in consumer electronics, vehicles, healthcare, defense, and communications. As their performance has improved, MEMS sensors have also found applications in oil exploration and geophysical studies. Pressure and temperature measurements during hydraulic fracturing have long been employed to improve downhole conductivity during oil and gas extraction. Nevertheless, the development of high-precision MEMS sensors for oil exploration remains an active area of research. This paper presents the design, fabrication, packaging, and characterization of a silicon-on-insulator (SOI) MEMS piezoresistive pressure sensor integrated with a temperature sensor. It also describes the design of a chamber intended to emulate conditions at the bottom of oil exploration wells. The sensors were successfully designed and fabricated on the basis of physics-based simulations, deep reactive ion etching and anodic bonding. The pressure sensors, together with the signal-conditioning system, exhibited a linear response with a sensitivity of 0.0268 mV/V/MPa and maximum hysteresis of 5.3%.

## 1. Introduction

Monitoring hydraulic fracturing is essential for the development of unconventional oil and gas resources [1,2]. This process involves injecting a high-pressure fluid into the target reservoir, thereby generating, extending, or connecting fractures to the natural fracture network. As a consequence, the porosity and permeability of underground reservoirs are enhanced, which increases the productivity of unconventional oil and gas resources. During hydraulic fracturing, the most critical downhole parameters are pressure and temperature. Pressure sensors used in hydrocarbon wells must withstand harsh operating conditions while maintaining accuracy, stability, and reliability over periods of several weeks. In particular, such sensors may be required to operate at pressures of up to 210 MPa and temperatures of approximately 200 °C [3].

Recent advances in microfabrication and micro-electro-mechanical systems (MEMS) have been successfully exploited in pressure-sensing technologies. Although substantial progress has been made in this area, there remains a clear need for MEMS-based solutions capable of meeting the demands of harsh environment applications [4,5,6]. The following subsections briefly review the principal sensing approaches, including optical fiber, resonant, capacitive, piezoelectric, and piezoresistive devices, together with temperature sensing strategies and the integration of pressure and temperature sensing within a single device.

### 1.1. Pressure Sensors

Various physical parameters, including acceleration, strain, humidity, and temperature, can be monitored using optical strain gauges or fiber optic sensors (FOS) [7,8,9]. Unlike conventional electrical sensors, FOS do not require an electrical supply, which makes them intrinsically passive and immune to electromagnetic interference [7,9]. Among the available options, one of the most reliable FOS approaches for downhole applications is based on the refractive index variation in a fiber Bragg grating (FBG). The FBG expands or contracts in response to the applied pressure or force applied to the FOS, thus altering the refractive index. However, the detection of slowly varying strain in the presence of temperature changes remains challenging because optical fibers are inherently temperature sensitive. Temperature compensation is therefore required in high-temperature and high-pressure measurements [10].

In resonant sensors, operation relies on exciting the mechanical resonant frequency. This frequency depends primarily on the spring constant of the bending section and on the effective mass, both of which vary with the applied load. Pressure can therefore be inferred from changes in resonant frequency [11,12]. To achieve the desired sensitivity and resolution, a high quality factor is required [13]. MEMS resonators offer low power consumption and the ability to operate at high frequencies with good stability and precision. They can also function across a wide pressure range, which makes them attractive for industrial applications. However, their performance and reliability may be affected by environmental disturbances, such as temperature fluctuations and mechanical shock. In addition, the fabrication of MEMS resonators can be complex and costly, which may limit their broader adoption.

Capacitive pressure sensors are generally implemented as parallel plate capacitors with a pressure-sensitive membrane on one or both sides. When pressure is applied, membrane deformation modifies either the electrode separation or the dielectric constant of the medium between the plates, producing a measurable capacitance change. Such sensors can operate over a wide pressure range, from approximately 250 Pa to 50 MPa [14], and they offer high sensitivity, low power consumption and improved temperature stability relative to piezoresistive or resonant counterparts [15]. In practice, however, measurements may be affected by parasitic and stray capacitances, as well as by variations in the dielectric properties of the medium, which complicates capacitance extraction and output signal linearization. As a result, their precision may be degraded. Only a limited number of capacitive pressure sensors have reported precision values exceeding 0.05% full scale (FS). In addition, fabrication typically requires essential pretreatment, sophisticated equipment and elevated process temperatures [16].

Piezoelectric sensors convert mechanical stress into an electrical signal [17,18]. When pressure is applied, the piezoelectric crystal structure deforms, which modifies the internal dipole moment of the piezoelectric material and changes the surface electric potential. Because of this mechanism, piezoelectric sensors can operate with very low energy consumption and are effectively self-powered. Piezoelectric ceramics such as PZT, ${\mathrm{BiFeO}}_{3}$, ZnO, and AlN are widely used owing to their favourable piezoelectric properties [19]. These sensors can cover a wide pressure range, typically from 0.7 kPa to 70 MPa, and are known for high sensitivity, accuracy and robustness [20]. Their electronic interface, however, is more complex than that of many other sensor types. A charge amplifier is required to convert the high-impedance charge output into a voltage signal, and this amplifier must be located close to the sensing element [20].

The earliest and still one of the most widely used classes of pressure sensor is the piezoresistive sensor. In these devices, resistive elements are deposited, implanted or diffused onto a stress-sensitive membrane [5,21,22,23,24]. Variations in the resistance of the piezoresistors caused by membrane stress are converted into a voltage output through the piezoresistive effect. Materials such as single-crystal silicon, polysilicon and ${\mathrm{Si}}_{3}N_{4}$ are frequently used in pressure-sensitive diaphragms. Silicon-based piezoresistive sensors are particularly attractive because of their fabrication simplicity, good sensitivity, high linearity and straightforward signal processing [23]. Their principal drawbacks are relatively high power consumption and temperature dependence, which typically require electronic compensation.

### 1.2. Temperature Sensors

A range of MEMS temperature sensors has also been developed, including capacitive, resonant, piezoresistive and resistor-based types. Capacitive temperature sensors typically comprise two plates or comb-drive structures whose spacing or overlap changes with temperature [25]. Resonant temperature sensors rely on temperature-dependent frequency shifts caused by thermal strain, which arises primarily from differences in the thermal expansion coefficients of the substrate and device layers [26]. Piezoresistive temperature sensors operate through stress-induced resistance changes in embedded piezoresistors; in this case, the stress is generated by mismatches in the thermal expansion coefficients of the constituent materials [27,28]. Resistor-based temperature sensors use a temperature-dependent resistor or thermistor, exploiting the principle that the electrical resistance of certain materials changes predictably with temperature [29].

### 1.3. Integration of Temperature and Pressure Sensors

The integration of pressure and temperature sensing within MEMS devices at the device level is now relatively mature and is important for further miniaturization, intelligent sensing and multi-parameter monitoring. In [12], a resonant differential-pressure sensor was developed on a single silicon-on-insulator (SOI) wafer, incorporating two resonators for pressure sensing, a platinum resistor for temperature sensing and an H-shaped resonator for static-pressure compensation. By contrast, [23] proposed a vertically integrated solution based on flip-chip bonding and through-silicon vias, allowing the pressure and temperature elements to be fabricated on separate wafers and optimized independently. In [24], the parasitic temperature sensitivity of a single piezoresistive pressure sensor was exploited to extract pressure and temperature simultaneously through bivariate polynomial calibration. Although those studies all employed platinum resistors for temperature sensing, ref. [28] combined capacitive pressure sensing with a metal resistor used as a temperature sensor.

In the present work, an integrated pressure and temperature sensor was designed and fabricated to monitor conditions at the bottom of oil exploration wells during hydraulic fracturing. The device was intended to operate reliably over a temperature range of 90 to 115 °C and at pressures of up to 70 MPa. In addition, packaging constraints required the total die assembly to remain within approximately 2 mm per side. On the basis of these specifications and the preceding analysis, piezoresistive pressure sensing and resistive temperature sensing were selected. An SOI wafer was chosen in order to improve thermal stability. To the best of our knowledge, this is the first report of an integrated pressure temperature sensor specifically intended for downhole applications. The novelty of the design lies in the realization of both piezoresistors and temperature resistors without an ion-implantation step, together with a process that enables a direct electrical connection from the top surface of the device layer to the handle wafer. This connection facilitates the current flow required during anodic bonding to form the sealed pressure-sensor cavity.

## 2. Materials and Methods

This section describes the design and fabrication of piezoresistive pressure sensors and resistive temperature sensors. First, the overall MEMS structure is introduced. The pressure-sensor design is then discussed, together with the associated finite element modeling. This is followed by the design and finite element modeling of the temperature sensor. Finally, the fabrication sequence is described in detail.

### 2.1. MEMS Sensors Structure

The MEMS structure was fabricated using a standard microfabrication process based on SOI technology. This approach allows greater flexibility in the selection of the active layer resistivity, which helps to define the piezoresistive elements more precisely and facilitates the formation of ohmic contacts, thereby eliminating the need for an ion implantation [30]. The active layer was isolated from the handle wafer by a buried oxide (BOX) layer of 2 μm of thickness. This active layer was micromachined to define both the piezoresistive pressure sensors and the resistive temperature sensors. A membrane was formed in the handle wafer by etching the bottom side of the SOI wafer, with the membrane connected to the active layer through the BOX layer.

### 2.2. Design and Simulation of Pressure Sensor

**Figure 1:**
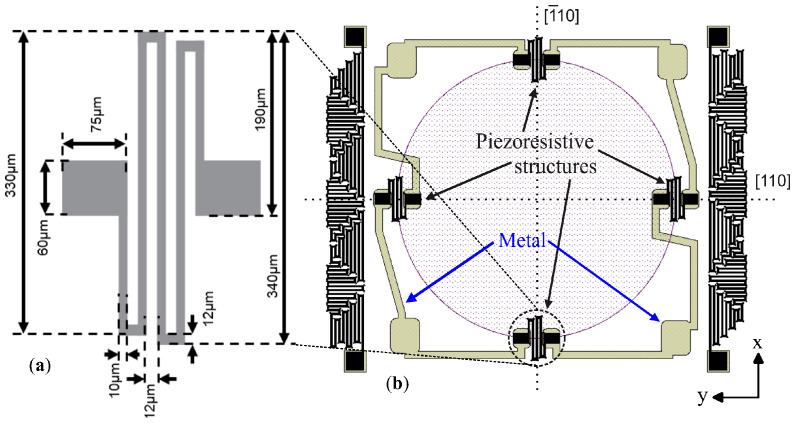
Layout design of (**a**) the serpentine-shaped piezoresistive elements and their dimensions, and (**b**) the location of the piezoresistive elements on top of the membrane.

The membrane geometry is a critical design parameter in pressure sensors. Rectangular membranes tend to exhibit maximum stress at the center of each side of the perimeter. In the present work, a circular membrane was selected because it provides a more uniform stress and strain distribution along the perimeter, as illustrated in Figure 1. For membranes of equivalent area and thickness, the strain in a circular membrane is lower than the maximum stress typically observed in a rectangular membrane. A membrane-isolated cavity was formed by partially machining the handle side of the SOI wafer using the Bosch deep reactive ion etching (DRIE) process, followed by wafer bonding to a Borofloat 7740 wafer to obtain a sealed enclosure. A specific set of structures was required on the device layer of the SOI wafer to enable the anodic bonding stage.

To exploit the piezoresistive effect effectively, the orientation of the piezoresistors must be selected carefully. It is well established that p-type silicon exhibits a strong piezoresistive effect when aligned along the [110] direction [31,32]. When stress is applied in this direction, the valence bands, including the light and heavy hole bands, split and warp, enhancing carrier mobility and producing substantial changes in resistivity. Accordingly, SOI wafers with a (100) plane, a 5 μm thick p-type device layer of resistivity 0.015 Ω-cm, and a 450 μm thick handle wafer were selected for the pressure sensor. Anisotropic chemical etching with potassium hydroxide (KOH) was used to define the piezoresistive structures from the active layer down to the BOX layer. The non-vertical profile produced by KOH etching results from the intersection of the (111) and (100) planes. This inclined profile also facilitates the continuity of the metal lines from the top (100) surface, across the etched (111) plane, and down to the BOX layer, which lies parallel to the (100) plane.

Finite element simulations were carried out in COMSOL Multiphysics version 4.0 [33] to examine the influence of the silicon structure parameters on the surface stress experienced by the piezoresistive elements. The mechanical response of the membrane was evaluated numerically by applying pressure to the device layer side of the sensor and using a Young’s modulus of E=129 GP and a Poisson ratio ν=0.28 for silicon [34]. Multiple finite element models covering a range of membrane thicknesses and diameters were analyzed in order to select dimensions that kept the stress below the silicon yield strength of 7.7 GPa [35].

First, serpentine-shaped piezoresistive sensing elements were designed following the resistive rosette model proposed in [36,37], as shown in Figure 1a. Four piezoresistors were then defined along the radial and circumferential axes of the membrane, aligned with the [110] direction and positioned near the membrane edge, as shown in Figure 1b. In this configuration, the resistance of the two longitudinal piezoresistors increases because of the positive piezoresistive coefficient, whereas that of the two transverse piezoresistors decreases because of the negative coefficient when pressure is applied to the membrane [36,37].

### 2.3. Design and Simulation of Temperature Sensor

**Figure 2:**
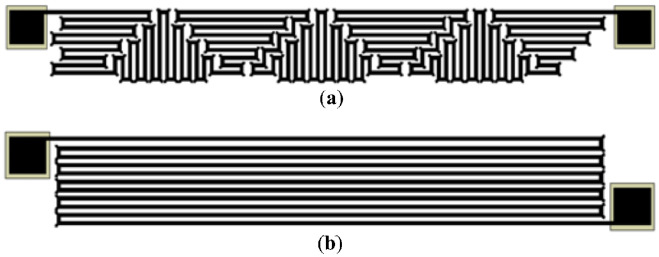
Layout of the studied temperature sensors. (**a**) Longitudinal and transverse piezoresistive sections for canceling the piezoresistive contribution due to the stress, and (**b**) only longitudinal sections.

The temperature sensors were positioned in regions of low mechanical stress so that their response would be dominated by temperature rather than strain. Owing to the die size constraints, these resistors could not be placed far from the membrane edge. Figure 1b shows the proposed layout intended to minimize piezoresistive contributions when the temperature resistors are integrated close to the membrane of the pressure sensor. The design was evaluated by finite element simulation of two resistor layouts, as shown in Figure 2. One layout used a simple serpentine with all segments aligned along the [110] crystal direction (see Figure 2b), whereas the other used the proposed mixed serpentine design (see Figure 2a). The proposed combines segments arranged both parallel and perpendicular to the [110] direction, thereby forming a mixed serpentine shape. In this configuration, individual segments experience positive and negative resistance changes caused by strain outside the membrane perimeter, as illustrated in Figure 2a. As a result, the piezoresistive contributions of the different segments tend to cancel one another [38]. An additional advantage of this layout is the more efficient use of the available die area.

### 2.4. Fabrication of Sensors

**Figure 3:**
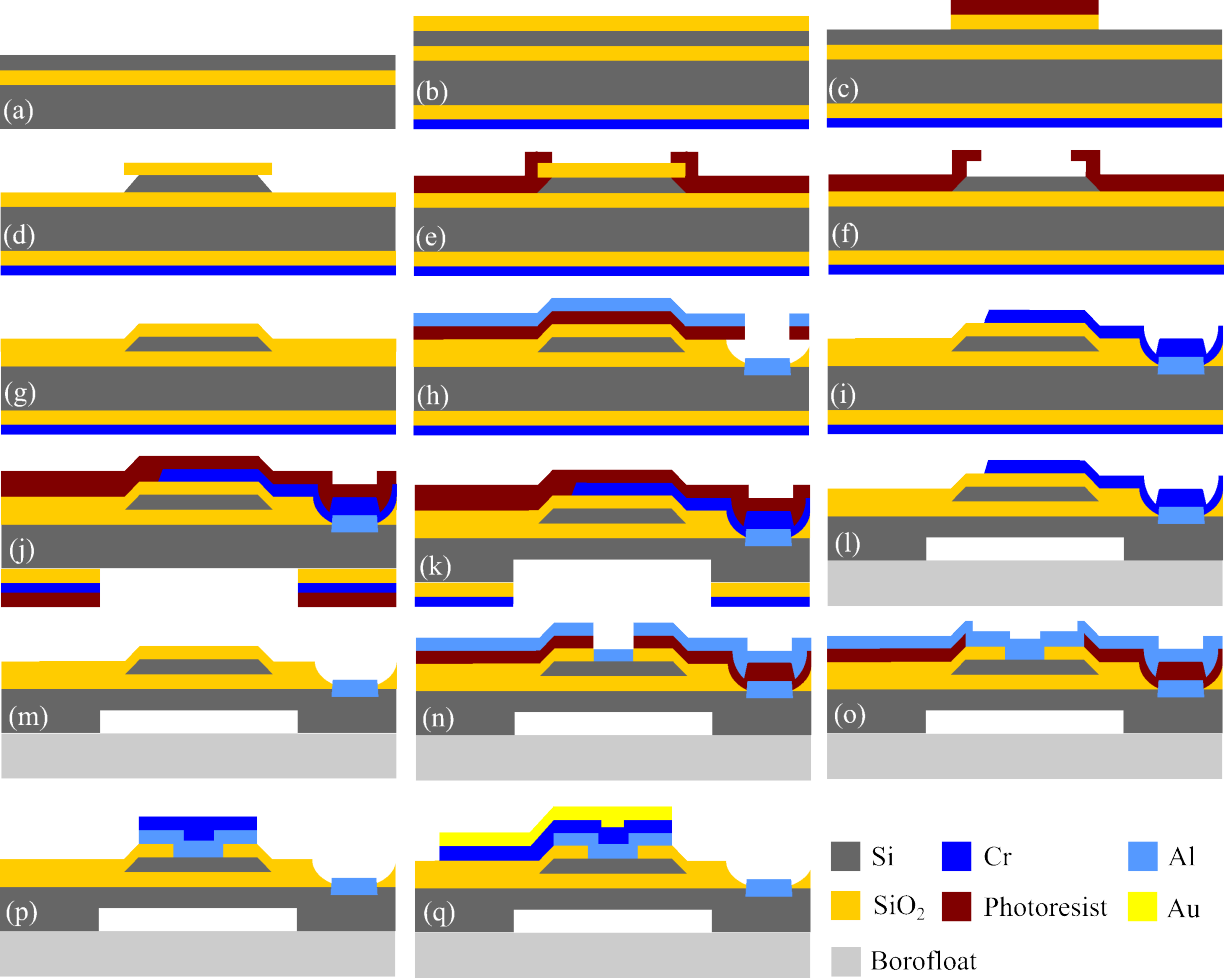
Fabrication steps for MEMS pressure and temperature sensors.

This subsection summarizes the fabrication sequence for the pressure and temperature sensors. The process flow is illustrated in Figure 3 and comprises the following steps:

(a) Start with a 4-inch SOI wafer consisting of a 5 μm device layer (100) plane with a resistivity of 0.015 Ω-cm, a ${\mathrm{SiO}}_{2}$ BOX layer, and a 450 μm handle wafer with a resistivity of 10 Ω-cm.
(b) The SOI wafer was subjected to a dry oxidation for 30 min at 1000 °C to grow 110 nm of ${\mathrm{SiO}}_{2}$ on both sides. Then, a layer of 100 nm of chrome was deposited on the bottom of the wafer by sputtering, using a rate of 0.75 Å per second for the first 25 nm, and 1.0 Å per second thereafter, for a total deposited thickness of 60 nm.
(c) Structures were defined on the device layer using Shipley S1813 photoresist. These structures include the piezoresistive pressure sensors, the resistive temperature sensors and the support structures required for the later wafer bonding stage described in step (l). A buffered oxide etch (BOE) was used to etch the top oxide while leaving the defined structures protected by photoresist and oxide.
(d) The top device layer was etched with KOH at 82 °C until the BOX layer was reached. A slightly modified mask, combining triangular and rectangular structures, was used relative to that reported in [37].
(e) A thick layer of photoresist was deposited and patterned with narrowed (undersized) features relative to the device mask design, leaving only narrow openings above the device structures. This protects the upper edges and exposed sidewalls produced by the KOH etch.
(f) The previously deposited photoresist was used as a protective layer during the BOE of ${\mathrm{SiO}}_{2}$. In particular, it protects the bottom BOX layer at the oxide interface with the KOH etched sidewalls of the device structures.
(g) After stripping the photoresist, etch the bottom chromium, and clean the wafer in piranha solution. A second dry oxidation step to grow 80 nm of ${\mathrm{SiO}}_{2}$ was conducted. Subsequently, 100 nm of chromium was deposited on the bottom of the wafer by sputtering, initially at 0.75 Å per second for the first 25 nm and then at 1.0 Å per second for the remaining 75 nm.
(h) A sacrificial mask on the device side was defined by photoresist patterning with narrow openings that allow the BOE in the BOX layer. The geometry of these openings is critical for the subsequent anodic-bonding process [39]. Because the BOE is isotropic, an undercut profile is formed in the BOX layer beneath the photoresist openings. Sixty nm of aluminum was deposited by sputtering on the device side, allowing metal to reach selected regions of the handle wafer through the BOX openings.
(i) After cleaning the photoresist, the top metal was lifted-off, leaving aluminum only within the BOX openings. Then, a second lift-off process on the device layer was performed to define a sacrificial metal layer of 200 nm of chromium by sputtering. This metal provides a continuous connection between the top of the device structures and the handle wafer through the BOX openings. The continuity is maintained from the top surfaces of the device structures, across the micromachined sidewalls, on to the top of the BOX layer and through the smoothly isotropic BOE profile down to the handle wafer.
(j) Both sides of the SOI wafer are coated with photoresist. The photoresist on the handle wafer side is patterned, and the chromium and oxide layers are etched on that side.
(k) The pressure sensor cavity is defined by partially etching the handle wafer with DRIE. Each DRIE cycle consisted of a passivation step followed by an etching step. The passivation step uses 76 sccm of $C_{4}F_{8}$, 8 W of forward RF power, and 805 W of inductively coupled plasma (ICP) power for 5 s. The etch step uses 76 sccm of ${\mathrm{SF}}_{6}$, 27 W of forward RF power, and 765 W of ICP power for 5 s. The average etch depth per cycle is 0.3 μm of silicon. This process defines the pressure sensor membrane with a final thickness of 190 μm. The total anisotropic etch depth in the handle wafer was 260 μm, requiring approximately 145 min of DRIE processing. Residual photoresist is then removed by dry $O_{2}$ etching, followed by wet etching of the chromium and oxide layers on the handle wafer.
(l) The bottom chromium layer on the handle wafer was wet etched, followed by a BOE of the handle oxide layer. The photoresist on the device side was stripped, and the wafer was cleaned with Piranha solution before the anodic bonding of the SOI wafer to the Borofloat wafer. The metallized top layer acts as an electrode for the surrounding contacts in the pressure cavity, ensuring the current flow required during the anodic bonding process at the interface between the handle wafer and the glass wafer. The anodic bonding was carried out in an SB6/8 wafer bonder using a membrane pressure of 150 kPa, a vacuum of $6\times 10^{-5}$ mBar, a temperature of 280 °C, a voltage of 1000 V, and a maximum current of 12 mA. During bonding, the current remained constant at 12 mA for 100 s and then decayed exponentially to 240 μA after 7 min and 23 s.
(m) After bonding, the sacrificial top chrome contacts were etched to prepare for the formation of ohmic contacts on the top surface of the device structures, particularly the piezoresistive sensors.
(n) A lift-off photoresist to define openings for ohmic contacts on the device layer of both the pressure and temperature sensors was used. Then 75 nm of Al/Si (95% and 5%, respectively) was deposited at 164 °C by sputtering. The use of Al/Si promotes diffusion into silicon and facilitates ohmic contact formation.
(o) After cleaning the lift-off photoresist, a new lift-off photoresist was applied to define wider openings for the ohmic contact regions on the top of the sensors. Subsequently, a second 75 nm of Al/Si (95% and 5%, respectively) layer is deposited at 174 °C by sputtering.
(p) Fifty nm of chromium was deposited by sputtering to protect the top Al/Si interface temporarily from oxidation. The lift-off photoresist was removed, and the bonded wafers were cleaned in piranha solution. Ohmic contacts were then formed by thermal treatment in an inert atmosphere at 450 °C for 30 min.
(q) After ohmic-contact formation, the oxidized top chromium was removed. Using lift-off photoresist, a fresh 75 nm chromium layer was deposited by sputtering, followed by 250 nm of gold by evaporation on the device layer, and complete the final lift-off step. Chromium and gold were deposited continuously from the top of the device structures across the machined sidewalls, thereby defining the metallized interconnections of a full Wheatstone bridge. The same metallization is used to form the wire-bond pads after wafer dicing. The dicing process begins by partially cutting the Borofloat wafer from the bottom.

## 3. Results

**Figure 4:**
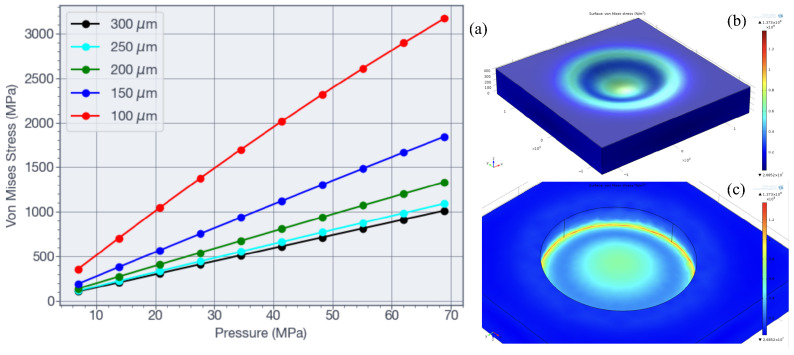
Finite element simulation results for the membrane. (**a**) Maximum von Mises stress for various membrane thicknesses as a function of applied pressure. (**b**) Top surface and (**c**) bottom surface von Mises stress distributions for a circular membrane with a diameter of 1550 μm, a thickness of 190 μm, and applied pressure of 70 MPa.

A three-dimensional model of the MEMS structure was developed in COMSOL. Figure 4 shows the membrane simulation results. Figure 4a shows the von Mises stress as a function of the applied pressure in the range of 7 MPa to 70 MPa for membrane thicknesses of 100, 150, 200, 250, and 300 μm, with a membrane diameter of 1550 μm. Based on these results, a membrane thickness of 190 μm was selected, corresponding to a von Mises stress of approximately 20% of the fracture strength of silicon. For the selected membrane dimensions, Figure 4b,c show the von Mises stress distribution on the upper and lower membrane surfaces under an applied pressure of 70 MPa.

**Figure 5:**
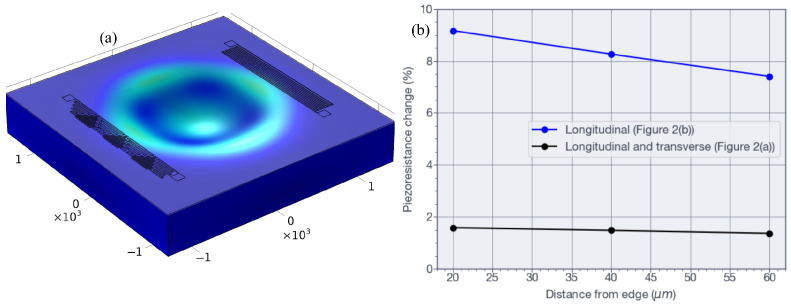
Finite element simulation of the temperature resistors. (**a**) Membrane under 70 MPa of pressure with the resistor structures shown in Figure 2a,b. (**b**) Comparison of the percentage of change in the piezoresistance between both designs due to membrane stress as a function of distance from the membrane edge.

Figure 5 shows the simulation results for the temperature resistor structures depicted in Figure 2a,b. Figure 5a shows the von Mises stress distribution under an applied pressure of 70 MPa at a constant temperature, with both resistor structures positioned 20 μm from the membrane edge. Figure 5b compares the relative resistance change of the two resistor configurations under an applied pressure of 70 MPa at the center of the membrane.

**Figure 6:**
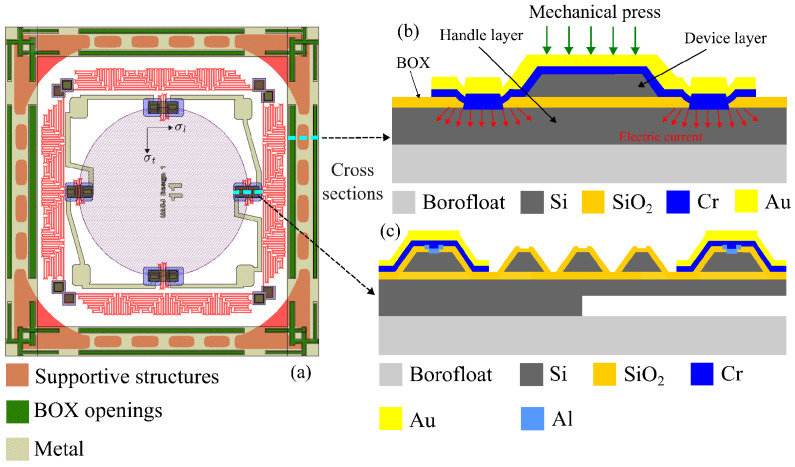
(**a**) Complete layout design, including the four pressure piezoresistors and four temperature resistors. (**b**) Cross-section of the supportive structure showing the BOX openings. (**c**) Cross-section of the piezoresistor and bonding support structure.

The complete MEMS design, including the support structures, BOX openings, and metal layers, is shown in Figure 6. Figure 6a shows the layout with four pressure piezoresistors and four temperature resistors. The cross-section of the support structure is shown in Figure 6b. As shown there, the metal layer was deposited along the inclined edges produced by KOH etching, thereby connecting the device layer to the handle wafer. Figure 6c shows cross-sections of the pressure sensor and the bonding support structure, located at the top left and top right of the layout, respectively.

**Figure 7:**
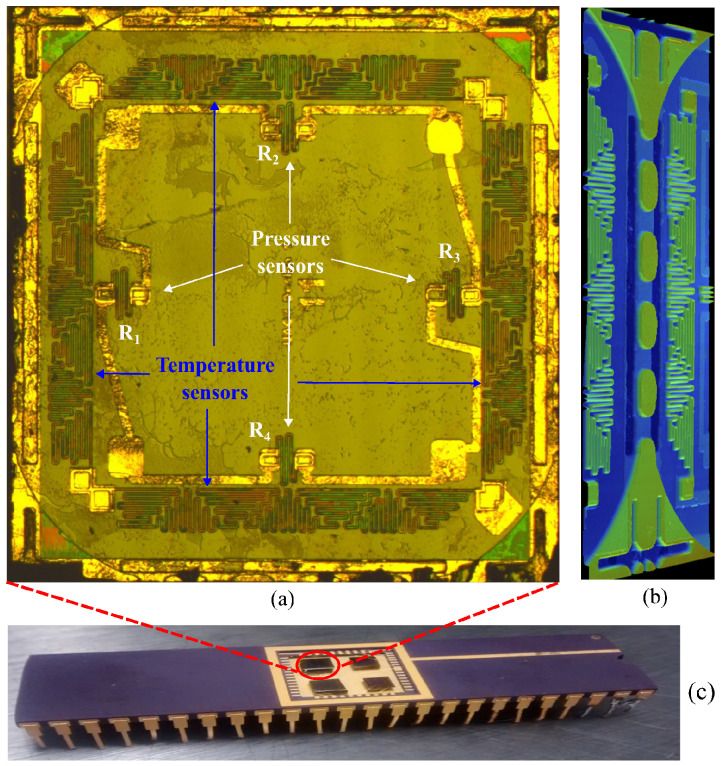
Fabricated MEMS sensor. (**a**) Top view of the pressure and temperature sensors. (**b**) Perfilometer image showing the support structure used for bonding the SOI and Borofloat wafers. (**c**) Four dies wire bonded in a 48-pin dual in-line package.

The fabricated MEMS sensor, which includes four pressure sensors arranged as a full Wheatstone bridge and four temperature sensors, is shown in Figure 7. Figure 7a shows a top view of the MEMS structures and their support features. Figure 7b depicts a perfilometer image, acquired with a Dektak Bruker perfilometer, of the support structure between two adjacent dies. The MEMS sensor chips were attached from the bottom with silver paint and wire-bonded in a 48-pin dual in-line package, with four dies per package, as shown in Figure 7c.

**Figure 8:**
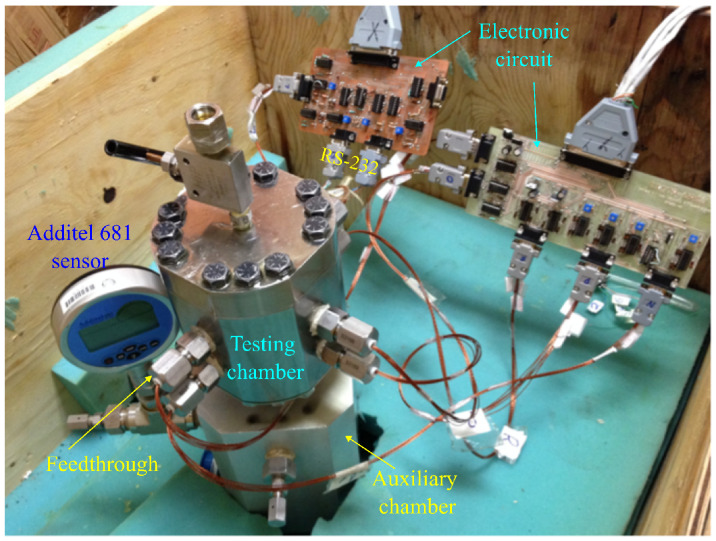
Low-cost setup used for the characterization of the pressure and temperature sensors.

An experimental arrangement was developed to characterize the pressure and temperature sensors and to validate the proposed design. The setup used for pressure measurements at different temperatures is shown in Figure 8. A chamber for high-pressure testing was developed for the piezoresistive sensors. The design comprised a low-cost, three-stage pressure system: (i) a manual deadweight tester (DWT1305D-100); (ii) a Simplex RC-series piston cylinder connected to an auxiliary chamber designed to separate the test fluids (see Figure 8); and (iii) a custom stainless-steel chamber equipped with eight Conax HPPL14 high-pressure feedthroughs, each carrying six AWG 24 electrical cables (see Figure 8). This three-stage system enabled the separation of the specialized hydraulic fluid utilized by the deadweight tester from the oil under test with MEMS sensors. It also facilitated the testing of high-pressure and temperature sensors using up to 48 independent electrical lines. All of the designed chambers complied with autoclave high-pressure connection standards. System pressure was monitored continuously through RS-232 communication with an Additel 681-series high-precision pressure gauge. The interconnected chambers, feedthroughs and signal-processing electronics are also shown in Figure 8.

**Figure 9:**
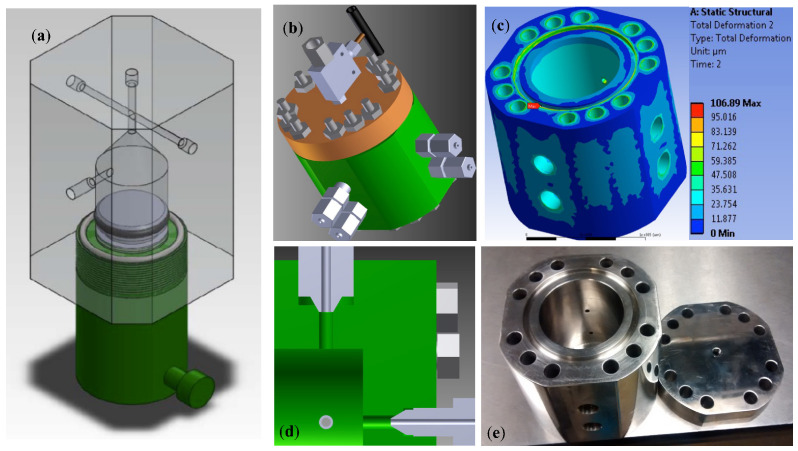
Custom-designed chambers for high-pressure testing. (**a**) Simplex piston cylinder for fluid isolation beneath the auxiliary chamber. (**b**) High-pressure test chamber showing four of eight feedthrough connectors. (**c**) Finite element simulation of the stainless steel test chamber. (**d**) Cross-section of the test chamber showing autoclave high-pressure connectors. (**e**) Machined test chamber showing the internal cavity for sensor placement.

Figure 9 shows the stainless-steel chamber design. The auxiliary chamber in Figure 9a, used as a fluid separator, and the test chamber in Figure 9b–e were both designed to withstand 70 MPa, on the basis of finite element simulations with a safety factor of 4. The upper chamber in Figure 9a corresponds to the lower chamber shown in Figure 8. A Simplex piston was attached beneath this chamber. Pressure from the deadweight tester was applied to the piston, which in turn pressurized the test oil in the auxiliary chamber. This pressurized oil was connected to the test chamber positioned above it, as shown in Figure 8. The test chamber had a height of 4.8 in, an outer diameter of 6 in, an inner diameter of 3 in, and an internal depth of 3.5 in to facilitate sensor placement and testing. In the finite element simulation of the stainless-steel chamber, shown in Figure 9c, the maximum von Mises stress was less than 20% of the tensile yield strength of 2.1 GPa for the American Iron and Steel Institute (AISI) 302 austenitic stainless steel [30].

As illustrated in Figure 6a, four piezoresistors were aligned along the radial and circumferential axes of the membrane in the [110] direction. Positioned at the membrane edge, they were connected in a full Wheatstone bridge to measure the piezoresistive response of the pressure sensor. For the layout shown in Figure 6a and a device layer resistivity of 0.015 Ω-cm, each serpentine piezoresistor had a zero-pressure resistance of $R_{o}=250\;\Omega$. Figure 6a also indicates the directions of the longitudinal stress $\sigma_{l}$ and transverse stress $\sigma_{t}$. Piezoresistors $R_{1}$ and $R_{3}$ are subjected to stress orientations rotated by 90 degrees with respect to those affecting $R_{2}$ and $R_{4}$. Consequently, the longitudinal stress component for $R_{1}$ and $R_{3}$ corresponds to the transverse stress component for $R_{2}$ and $R_{4}$, and vice versa. The relationship between resistance change and piezoresistive response for each piezoresistor can be written as

(1) $$\begin{array}{cc} \frac{\Delta R}{R} & =\pi_{l}\sigma_{l}+\pi_{t}\sigma_{t}\\ \; & =\left(\pi_{l}+\nu \pi_{t}\right)\sigma_{t}\\ \; & =X, \end{array}$$

where ν is the Poisson ratio of silicon, and $\pi_{l}$ and $\pi_{t}$ are the longitudinal and transverse piezoresistive coefficients, respectively.

**Figure 10:**
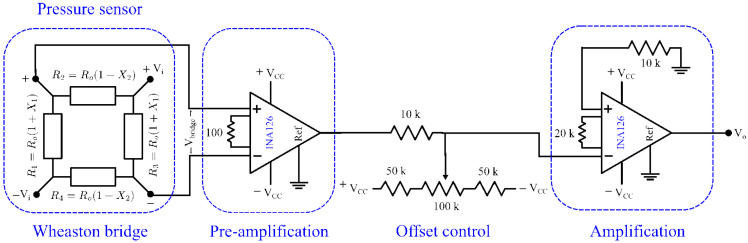
Electronic circuit used for calibration and readout of the pressure sensor signals.

As shown in Figure 8, an electronic circuit was used to ensure accurate calibration of the sensor signals. The schematic is shown in Figure 10. $X_{1}$ and $X_{2}$ represent the relative resistance changes of the piezoresistive elements, as defined by Equation (1). At zero pressure, the resistances are equal, such that $R_{o}=R_{1}=R_{2}=R_{3}=R_{4}$. The input-output relationship of the Wheatstone bridge is expressed by

(2) $$\begin{array}{cc} \frac{V_{o}}{V_{i}} & =\frac{R_{1}R_{3}-R_{2}R_{4}}{\left(R_{1}+R_{2}\right)\left(R_{3}+R_{4}\right)}\\ \; & ≅\frac{X_{1}+X_{2}}{2\left(1+X_{1}-X_{2}\right)}\\ \; & =\sigma_{l}\frac{\left(1-\nu \right)\pi_{l}+\left(\nu -1\right)\pi_{t}}{2}. \end{array}$$

**Figure 11:**
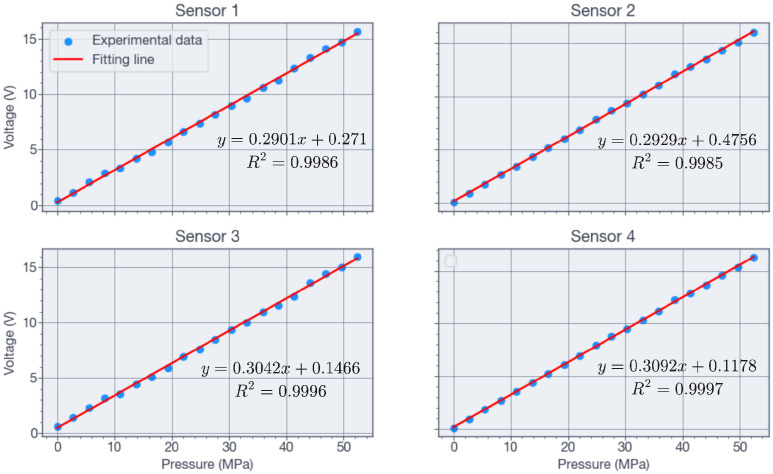
Output voltage of the pressure sensor after signal conditioning and amplification as a function of pressure.

The readout circuit incorporated INA2126 instrumentation amplifiers, a voltage supply of $V_{i}=2$ V, and stages for pre-amplification, offset adjustment, and amplification. The total gain, *G*, was 7245. Because in practice $X_{1}$ and $X_{2}\ll 1$, the Wheatstone bridge exhibits only slight non-linearity, as shown in Figure 11 for four sensors tested up to 55 MP. From Equation (2), the sensor sensitivity is

(3) $$S=\frac{\left(1-\nu \right)\pi_{l}+\left(\nu -1\right)\pi_{t}}{2}.$$

The Wheatstone bridge calibration and overall circuit response can be expressed as

(4) $$V_{o}=GV_{bridge}+V_{off}$$

where *G* is the total gain of the conditioning stages shown in Figure 10, $V_{bridge}=V_{i}SP$, and $V_{off}$ is the offset voltage. Consequently, the pressure is expressed as $P=\frac{V_{o}-V_{off}}{GSV_{i}}$, and the nominal conversion factor can be defined as $M=\frac{1}{GSV_{in}}$. Thus, *P* is given by

(5) $$P=M(V_{o}-V_{off})$$

To account for discrepancies between the nominal and actual scale factors, the conversion factor was adjusted experimentally using a reference pressure point $P_{ref}$.

(6) $$M_{calib}=\frac{P_{ref}}{V_{o,ref}-V_{off}}$$

where $V_{o,ref}$ is the output voltage at $P=P_{ref}$. The final pressure estimate is

(7) $$P=M_{calib}\left(V_{o}-V_{off}\right)$$

During calibration, the pressure indicated by the Additel 681 gauge was used as the reference value. It was not assumed to be the true pressure; rather, it was treated as a traceable reference with an associated uncertainty. The *G* value was verified experimentally from the slope of the calibration curve relating $V_{o}$ with *P*. The measured value of $V_{o}$ at $P_{ref}=50$ Mpa was then compared with the value of *G* expected from the designed conditioning stages. The total calibration uncertainty was obtained by combining the uncertainty of the reference gauge with the repeatability, resolution, and fitting uncertainty of the proposed sensor system. Finally, the pressure resolution of the readout system was estimated as

(8) $$\Delta P_{res}=\frac{\Delta V_{o}}{GSV_{in}}$$

where $\Delta V_{o}$ is the minimum resolvable output voltage variation of the readout system.

The output voltage $V_{o}$ was measured using an Agilent 34401A 6$\frac{1}{2}$ digit digital multimeter operated on the 100 V DC range. According to the manufacturer’s specifications, this range provides a display resolution of 100 μV and a DC voltage accuracy of ±(0.0045% of reading + 0.0006% of range) under the one-year specification. The voltage uncertainty used in the pressure uncertainty analysis was estimated as $u\left(V_{o}\right)=\pm \left(0.0045\%V_{o}+0.0006\%100\;V\right)$. The estimated pressure resolution $\Delta P_{res}$ was $2.53\times 10^{-4}$ MPa.

**Figure 12:**
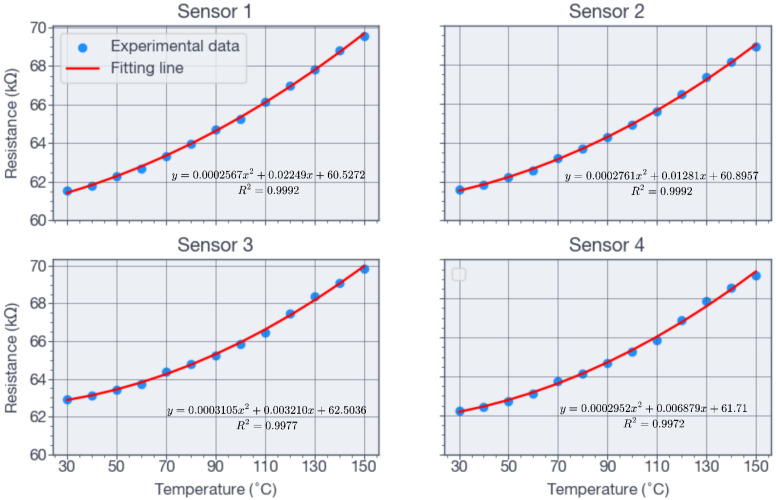
Resistance as a function of temperature for the four temperature sensors.

The temperature sensors were implemented to measure the operating temperature at the bottom of the well directly and to compensate for minor temperature-induced variations in the piezoresistive elements. As shown in Figure 6a and Figure 7a, each die incorporated four resistive temperature sensors. Two resistors were connected in series, and the resulting two branches were connected in parallel, with the resistance measured across opposite corners. All temperature tests were performed in a Barnstead Thermolyne 48000 electric furnace over the range of 30 °C to 150 °C, in steps of 10 °C. Figure 12 shows the measured resistance values for the four sensors, obtained with an Agilent 34401 meter. The resistance increased on average by 8.5% over the range of 32 °C to 140 °C. As shown in Figure 12, a quadratic approximation of the resistance as a function of temperature was obtained.

**Figure 13:**
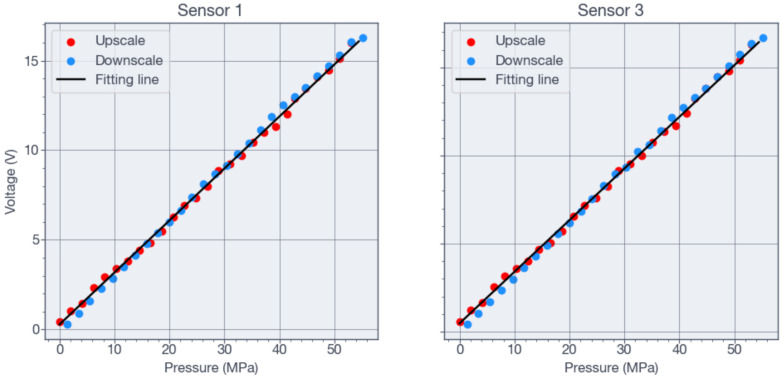
Hysteresis measurement showing the behavior of the sensor during upscale and downscale pressure cycles.

During pressure testing, hysteresis was observed in the pressure sensors. Figure 13 shows the upscale and downscale measurements of sensors 1 and 3 at 32 °C, used to determine hysteresis. In this context, hysteresis quantifies a measure of the lag or deviation between the increasing and decreasing pressure cycles, which is an important indicator of sensor performance and accuracy. The maximum hysteresis occurred at 40 MPa, with values of 4.5% and 5.3% FS for sensor 1 and sensor 3, respectively. Sensors 2 and 4 present similar results. Although hysteresis and time drift may originate from related physical mechanisms, this study did not systematically evaluate time drift. Therefore, no quantitative claim regarding long-term stability can be made at this time.

## 4. Discussion

The final MEMS pressure sensor design was selected on the basis of finite element results shown in Figure 4 and Figure 5. The structure employed a membrane with a diameter of 1550 μm and a thickness of 190 μm. At the maximum applied pressure of 70 MPa, the maximum displacement at the membrane center was 9.5 μm, as shown in Figure 4b,c. The maximum von Mises stress at the inner edge was 1418.81 MPa (see Figure 4a), which corresponds to approximately 20% of the yield strength of silicon. As illustrated in Figure 4b,c, the largest strain occurred near the membrane edge, where the four piezoresistors were located. The results in Figure 5 guided the placement of the temperature resistors shown in Figure 2a. For an applied pressure of 70 MPa at a constant temperature, Figure 5b indicates that the proposed design of Figure 2a reduces the apparent piezoresistive contribution in the temperature resistor to less than 1.6%, compared with 9.2% for the design shown in Figure 2b when the resistors are located 20 μm from the membrane edge. The proposed design is applicable to smaller die dimensions by positioning temperature sensors near the stress regions on the membrane edge because of its advantageous self-canceling piezoresistive effect.

Anodic bonding of the handle side of the SOI wafer to the Borofloat wafer, corresponding to step (l) in Figure 3, is a critical fabrication step. The preceding process steps were designed specifically to enable this bonding stage. In particular, the support structures defined in step (c), and the metallized path extending from their top surface to the handle wafer through the BOX openings created in step (h) are essential during bonding. This conductive path facilitates the required current flow, $I_{max}$, defined as the maximum bonding current density in mA/${\mathrm{cm}}^{2}$, which occurs during the first few seconds of bonding and strongly influences bond quality [39], as shown in Figure 6b. $I_{max}$ is directly related to the charge transported to the depletion layer at the silicon-dielectric bonding interface, and hence to the formation of a strong bond. The bonding process also requires a voltage sufficient to overcome a minimum activation energy of 0.97 eV [39]. The relatively thick dielectric layer of 2 μm in the SOI wafers impedes $I_{max}$ flow through the wafers, making it more difficult to achieve the activation conditions required for bonding the handle wafer to the Borofloat wafer. Although SOI to Borofloat bonding has been reported for BOX thickness of 380 nm [40], thicker BOX layers require an alternative strategy; otherwise, substantially longer bonding times may be needed [40]. Most anodic bonding systems operate with a limited current in the range of 5 mA to 15 mA for at least one minute to achieve the activation conditions required at 1000 V for 4-inch silicon and Borofloat wafers. For this reason, step (i) of the fabrication process included the deposition of a thin chromium layer in the BOX openings (see Figure 3). This layer is electrically connected to the top of the support structures and facilitates the required current flow during anodic bonding. The edge support structures also help the devices to withstand the mechanical load applied during bonding. Without them, the sensing structures could fracture under the applied force. Figure 7b shows four central support structures with BOX openings on their sides, flanked by two temperature sensors corresponding to two neighboring sensor devices. Larger structures with internal BOX openings can also be seen at the top and bottom. Figure 7a further shows the final dicing lines, which cut the support structures in half.

The characterization stage was limited by the Viton O-ring used in the test chamber, shown in Figure 8, which withstood a maximum pressure of 55 MPa. Consequently, the maximum test pressure corresponded to 79% intended design pressure. The results in Figure 11 show a maximum linear error of 4.95% at 7 MPa, decreasing to less than 3.5% at 24 MPa. These results were obtained under the assumption that the Additel pressure sensor (shown in Figure 8) provided an accurate reference reading and that the temperature remained constant at 32 °C. The voltage-pressure relationship of the pressure sensors was fitted accurately by the linear equations shown in Figure 11, while the resistance-temperature relationship was fitted accurately by the quadratic equations shown in Figure 12. For both the pressure and temperature sensors, the goodness of fit ($R^{2}$) is greater than 0.99, indicating excellent sensing characteristics. The measured full-scale bridge output was $V_{bridge}=2.95$ mV for $V_{i}=2$ V, corresponding to a sensitivity of 0.0268 mV/V/MPa.

To the best of our knowledge, the literature does not report integrated temperature-pressure sensors designed for pressures above 40 MPa. The studies summarized in Table 1 focus exclusively on high-pressure sensors, and two of them rely only on simulation data [3,22]. Relative to those studies, the integrated sensor presented here is notable for its compact size, good linearity and useful sensitivity, although its pressure range and temperature tolerance remain lower than those of some previously reported devices. The temperature sensor also exhibited a satisfactory response.

## 5. Conclusions

A high-sensitivity MEMS piezoresistive pressure sensor integrated with a resistance temperature sensor was successfully designed, fabricated and characterized for downhole applications. The device was intended to support hydraulic-fracturing monitoring and was therefore designed for operation at pressures of up to 70 MPa and temperatures of at least 90 °C. The piezoresistive elements were designed successfully by selecting an active-layer resistivity of 0.015 Ω-cm, thereby removing the need for ion implantation. The temperature resistors were placed successfully near the edge of the pressure-sensor membrane by exploiting the self-cancellation of piezoresistive effects in a combined longitudinal-transverse serpentine layout. The proposed fabrication process employs 4-inch SOI wafers together with DRIE and anodic bonding. Auxiliary structures on the device layer were essential for successful anodic bonding of the SOI and Borofloat wafers. A cost-effective high-pressure test system was developed, enabling sensor testing up to 55 MPa and 150 °C. The fabricated pressure sensors exhibited a linear response with a sensitivity of 0.0268 mV/V/MPa and a hysteresis of 5.3% FS. The temperature sensors showed a quadratic response, with a maximum resistance change of 8.5% at 140 °C. 

## Figures and Tables

**Table 1:** Performance parameters comparison with published literature.

References	Pressure Range	Temperature Tolerance	Membrane Thickness	Sensitivity
[3]	0–200 MPa (sim.)	Room temp.	20 μm	0.0877 mV/V/MPa
[5]	0–150 MPa	Up to 200 °C	350 μm	1.1126 mV/MPa
[22]	0–40 MPa (sim.)	>600 °C	50 μm	3.395 mV/V/MPa
[6]	0–120 MPa	−10–70 °C	470 μm	0.425 mV/V/MPa
This work	0–70 MPa	30–150 °C	190 μm	0.0268 mV/V/MPa

## Data Availability

The raw data supporting the conclusions of this article will be made available by the authors on request.

## Abbreviations

The following abbreviations are used in this manuscript:

MEMS	Micro-electro-mechanical systems
SOI	Silicon-on-insulator
FOS	Fiber optic sensors
FBG	Fiber Bragg grating
DRIE	Deep reactive ion etching
BOX	Buried oxide
KOH	Potassium hydroxide
BOE	Buffered oxide etch
ICP	Inductively coupled plasma
